# Evaluating efforts to advance equity and inclusion in academic medicine: Charting a path for transformative change‡

**DOI:** 10.1017/cts.2026.10237

**Published:** 2026-01-26

**Authors:** Ju-Hsin Chen, Emma K.T. Benn, Erin Brittain, Nihal E. Mohamed

**Affiliations:** 1 Center for Scientific Workforce Excellence and Advancement, Department of Population Health Science and Policy, Icahn School of Medicine at Mount Sinai, USA; 2 Center for Scientific Workforce Excellence and Advancement, Center for Biostatistics, Department of Population Health Science and Policy, Icahn School of Medicine at Mount Sinai, USA; 3 Center for Scientific Workforce Excellence and Advancement, Department of Urology, Icahn School of Medicine at Mount Sinai, USA

**Keywords:** Equity, inclusion, theory-driven evaluation, biomedical research institutions, mixed-methods analysis

## Abstract

Despite the critical role academic medical institutions play in promoting equity and inclusion, these efforts are often undermined by fragmented and siloed evaluation approaches, a lack of theory-driven models, and inadequate data-driven methodologies. This commentary critically examines the factors contributing to the persistent challenges in implementing and advancing equity and inclusion initiatives in the United States (U.S.), focusing on academic medical institutions in particular. It underscores the need for theory-guided frameworks to better structure and evaluate such initiatives and highlights existing gaps in accountability and data-driven decision-making. Moreover, it argues for an integrated approach that combines multidisciplinary strategies, promoting collaboration across different sectors to develop more comprehensive and sustainable solutions. Ultimately, effective evaluation – grounded in data, theory, and diverse perspectives – is essential for promoting equity and inclusion and addressing disparities in U.S. academic medical institutions. By addressing these structural and evaluative shortcomings, institutions can move towards more impactful and equitable outcomes for underrepresented groups in STEM fields and the biomedical workforce more broadly.

## Introduction

The challenges faced by underrepresented groups – including individuals from racially and ethnically underrepresented populations, persons with disabilities, those from economically or educationally disadvantaged backgrounds, and women[1] – in academic medical institutions in the United States (U.S.) are well-documented, revealing systemic barriers hindering research and career advancement [2–4]. These include inhospitable work climates, lower financial compensation, limited leadership opportunities, implicit bias, and structural racism [2–4]. The importance of addressing these inequities extends to clinical and translational research specifically, where diversity in the research workforce directly impacts the questions asked, populations studied, and translation of findings to diverse communities [5]. Collectively, these individual, institutional, and systemic challenges contribute to the “representation gap” in the science, technology, engineering, and mathematics (STEM) fields – an issue that affects innovation, the scope of research questions, methodologies, analysis, evaluation, and the equitable translation of research into healthcare delivery and health outcomes of different populations [6,7].

The most recent data from the National Science Foundation (NSF) and the National Center for Science and Engineering Statistics (NCSES) show that underrepresented racial and ethnic minorities (URMs) make up just 14% of the U.S. science and engineering (S&E) workforce, despite comprising 31% of the general population [8]. These representation gaps extend to research funding and leadership, where systemic inequities continue to persist. Black researchers receive significantly less NIH funding than their white counterparts, in part due to topic choice, with only 10% of grant applications submitted by Black scientists funded compared to 17% for white scientists [9,10]. Similarly, gender-based inequities remain entrenched across STEM fields, particularly in academic medicine. While women make up nearly 51% of the U.S. population, they continue to be underrepresented in key areas of the scientific workforce. As of 2019, 27% of U.S. medical school deans, 45% of senior associate deans, and only 25% of department chair positions are held by women [10–12]. Challenges in retention and persistent salary inequities, which are further compounded by race and ethnicity, continue to hinder equity in compensation and career advancement, underscoring the urgent need for institutional reform.

Addressing these gaps is vital for fostering equity and inclusion [13,14]. The NIH defines equity in academic medical institutions as the consistent and systematic treatment of all individuals fairly, justly, and impartially, while striving to emphasize the importance of identifying and eliminating barriers that have historically excluded marginalized and underrepresented groups from full participation in academic and professional environments [15]. In academic medicine, this could be achieved by ensuring equal access to resources, opportunities, and decision-making processes, regardless of race, ethnicity, gender, or other characteristics. According to the NIH 2023 report “*Wide Strategic Plan for Diversity, Equity, Inclusion, and Access(DEIA)*,” equity goes beyond equality by removing systemic inequalities and creating conditions that allow all individuals, particularly those from underrepresented groups, to thrive [15]. The NIH also defines inclusion in academic medical institutions as the intentional integration of diversity into institutions’ operations, culture, and mission to achieve excellence in research, education, and patient care. This definition emphasizes the importance of creating an environment where underrepresented groups are intentionally included and provided with necessary resources and support to thrive and advance. This holistic approach of combining equity and inclusion enhances the institution’s ability to ensure fair opportunities for success to all members while addressing complex health challenges through innovative research questions and solutions.

Throughout this commentary, we use “workforce” to encompass the full spectrum of individuals who contribute to academic medical institutions’ research, clinical, educational, and administrative missions. This includes faculty across all ranks, researchers and research staff, clinicians, postdoctoral fellows and trainees, students, administrative personnel, and institutional leadership. While the specific challenges and opportunities for advancing equity and inclusion may vary across these groups, the systemic barriers and evaluation strategies we discuss are broadly applicable to the diverse academic medical workforce.

Several challenges may limit the success of academic medical institutions’ initiatives to promote equity and inclusion. These challenges include:

### Fragmented or siloed approaches to evaluation

Evaluations are an essential tool to both document the successes of equity and inclusion initiatives and to highlight areas for improvement. However, many institutions adopt isolated or uncoordinated strategies resulting in duplicated efforts and inconsistent messaging, diminishing their overall impact.

This fragmentation is particularly foundational, as it exacerbates the other three challenges. Without coordinated evaluation structures, it becomes more difficult to produce actionable insights or drive sustainable change.

Furthermore, there is often insufficient institutional consensus on what constitutes success. Different stakeholders may prioritize competing metrics: demographic representation in hiring and retention rates, career advancement of underrepresented faculty, equitable distribution of resources and opportunities, inclusive institutional climate as measured by surveys, or research productivity and funding success across demographic groups. These misaligned success metrics complicate evaluation design, make cross-institutional comparison challenging, and can lead to fragmented findings and initiatives. The results may meet numerical targets yet still fall short of achieving true equity or inclusion.

### Absence of guiding theoretical framework or logic models

Logic models can be developed by evaluators, program staff, or institutional leaders, but are most effective when created collaboratively. Engaging individuals with diverse disciplinary backgrounds and perspectives in the design process ensures that the model reflects the multifaceted nature of equity and inclusion initiatives and supports more coherent and actionable evaluation strategies.

Without well-defined theoretical frameworks or logic models to guide the development and evaluation of equity and inclusion initiatives, institutions may struggle to structure their efforts effectively, leading to disjointed or incomplete approaches that do not fully address the complexities of these issues.

An additional challenge is the variability in evaluator training and expertise. Many professionals conducting equity and inclusion evaluations in academic medical centers have not received formal graduate-level training in program evaluation theory and methods. Evaluators may come from diverse disciplinary backgrounds – including social sciences, public health, institutional research, or data analytics – each bringing valuable perspectives and methodological approaches. The lack of standardized training in equity-focused evaluation can complicate efforts to develop shared evaluation frameworks and comparable findings.

### Inadequate data collection, tracking, and accountability

The lack of robust mechanisms to gather and track relevant data impairs the ability to measure progress, identify gaps, and hold stakeholders accountable. Without these methods and tools, institutions face significant challenges in assessing the effectiveness of their initiatives and addressing barriers to equity and inclusion.

### Insufficient data-driven evaluation

The absence of comprehensive data-driven evaluation for equity and inclusion efforts limits the scope of institutional understanding. Without systematic data collection and analysis, key insights may be missed, limiting the ability to identify existing gaps, measure impact, and refine strategies to foster inclusion.

In this commentary, we will explore these four factors in greater depth, examining their impact on institutional efforts and offering potential strategies for overcoming these obstacles. Our discussion is intended for multiple stakeholders invested in advancing equity and inclusion in academic medical centers: program evaluators who design and implement assessments, institutional leaders (including chief diversity officers, deans, department chairs, senior administrators, and advocates) who allocate resources and set strategic priorities, and faculty and staff who collaborate on equity initiatives. While evaluators are a primary audience, meaningful progress depends on collaboration across all administrative levels and functional units.

We define program evaluation as the systematic collection, analysis, and interpretation of data to assess the design, implementation, and effectiveness of targeted initiatives (in this case, those focused on equity and inclusion). Evaluation in academic medical centers takes many forms and occurs at multiple levels – from departmental assessments of mentoring programs to institution-wide analyses of recruitment and retention patterns. Evaluators include dedicated evaluation professionals, institutional research staff, faculty conducting assessment projects, human resources analysts, or members of diversity offices charged with tracking progress. Importantly, the structure and coordination of evaluation activities vary considerably across institutions. Some academic medical centers have centralized evaluation units with dedicated staff, while others rely on decentralized, ad hoc approaches where evaluation responsibilities are distributed across departments or embedded within other roles. This structural variation influences the scope, resources, and institutional influence available to those conducting equity and inclusion evaluations. We acknowledge this heterogeneity and aim to provide frameworks applicable across diverse organizational contexts.

The following sections will outline actionable plans to improve the coordination, assessment, and integration of institutional efforts to enhance equity and inclusion.

## Actionable plans to improve institutional efforts to enhance equity and inclusion

**Figure 1.**
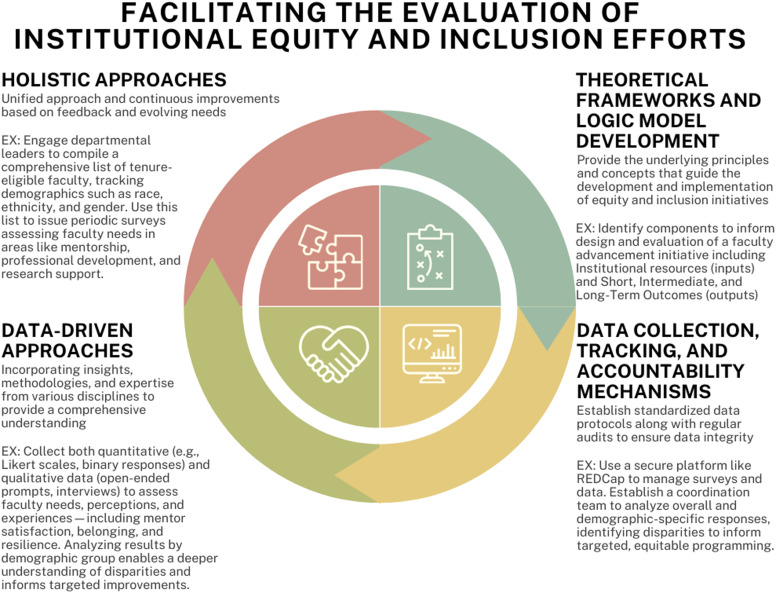
Major factors that affect the evaluation of equity-based efforts. Source: Developed by study team (self-generated).

There are major strategies that can be used to address the four factors contributing to the limited success of academic medical institutions’ initiatives to promote equity and inclusion (see Figure 1). These strategies include:

### The utility of holistic approaches to program evaluation

Fragmented or siloed approaches to evaluating academic and medical institutions’ efforts to improve equity and inclusion refer to initiatives within an organization that are disjointed, isolated, and lack coordination and accountability [6]. Without a shared strategy, there is limited integration of knowledge across units, which weakens the overall impact and reduces the potential for meaningful improvements in equity and inclusion efforts. Coordinated, cohesive evaluation processes are critical for fostering accountability and achieving consistent equity and inclusion goals across institutions [16].

Current research indicates that many equity and inclusion programs prioritize quantitative and descriptive measures, such as workforce demographic composition, over qualitative assessments of employees’ experiences and changes in work environment and culture [7]. This narrow focus on survey-based outcomes often overlooks crucial determinants of programs’ and efforts’ effectiveness, such as participants’ lived experiences and perceived impacts of these programs on the workplace [17]. In bridging evidence to action, evaluators play a critical role in systematically collecting and presenting data-driven insights, while advocates interpret these findings and communicate recommendations to institutional leaders to promote structural change.

Additionally, research indicates that focusing solely on short-term outcomes (i.e., those measured within the first year of program implementation) may not capture the full scope of these initiatives’ effects on institutional culture and practices [18]. The underutilization of long-term, longitudinal study designs (extending beyond one year post-implementation) limits the quality of outcome data, which is crucial for assessing the deeper, lasting impacts of equity and inclusion programs on both individuals and organizations. Furthermore, the lack of a standardized evaluation framework complicates efforts to compare findings and derive comprehensive insights [19]. This fragmentation can inhibit a clear understanding of program effectiveness and impede the overall progress of institutional equity initiatives.

Examples from other organizations’ efforts to enhance program evaluation, such as the National Aeronautics and Space Administration (NASA), provide valuable lessons. Over the past decade, NASA has made inclusion a central value by issuing strategic equity and inclusion plans, appointing a Chief Diversity Officer, and incorporating equity and inclusion objectives into performance evaluations for senior executives and supervisors. The organization also invested in an enterprise-wide database to track progress on these initiatives [19]. However, despite these comprehensive efforts, NASA’s workforce demographics have shown little change, with minimal growth in the representation of women and minorities, particularly in senior leadership positions. This highlights that while equity and inclusion initiatives have been implemented, they may still be functioning in isolated silos, lacking the necessary integration and coordination to produce widespread and lasting impact.

Thus, to effectively address the limitations of fragmented evaluation methods, researchers should adopt a comprehensive evaluation framework that integrates both quantitative and qualitative measures. This approach should assess equity and inclusion initiatives across multiple dimensions, such as demographic shifts, employee experiences, and organizational culture. For example, one strategy involves engaging departmental leaders to compile a centralized, comprehensive list of tenure-eligible faculty, tracking demographics such as race, ethnicity, and gender. This shared dataset can support coordinated surveys that assess faculty needs in mentorship, professional development, and research support, fostering a unified understanding of faculty development across the institution.

It is essential to promote collaboration and information sharing across departments and units, ensuring a unified approach to program evaluation and implementation. Moreover, ongoing and iterative evaluation processes should be implemented to track both short- and long-term program impacts. Continuous improvements can be made based on feedback and the evolving needs of the organization, leading to more effective and sustainable outcomes.

### The utility of theoretical frameworks and logic models to guide the development and evaluation of equity and inclusion programs

Developing effective programs and initiatives to enhance equity and inclusion in academic and medical institutions requires a deep understanding of theoretical frameworks and the construction of logic models [20]. Theoretical frameworks offer the foundational principles and concepts that guide the development and implementation of equity and inclusion initiatives [20]. Conversely, a logic model provides a visual representation of how these initiatives are expected to function, illustrating the connections among available institutional resources, planned activities, programs’ outputs, and outcomes in a coherent framework [21,22].

Prior programs on equity and inclusion have employed several theoretical models, including the Social-Ecological Model, the American Psychological Association’s (DEIA) Framework [23], and the National Institutes of Health (NIH) DEIA Framework [15]. These models elucidate factors influencing equity and inclusion programs development and evaluation at both micro (individual) and macro (organizational and societal) levels. They emphasize the importance of collaborative efforts to address significant barriers across four levels of influence: individual, professional, organizational, and societal, all of which affect program outcomes.

Logic models serve as diagnostic tools to identify existing needs and challenges, as well as to develop, implement, and evaluate programs or interventions. They graphically represent the relationships between available resources, program activities, and intended effects and outcomes, including the expected pathways of program operation, the contextual factors involved, and the desired changes. Importantly, logic models are not static; they should be periodically revised to reflect new evidence, lessons learned, and shifts in context, resources, activities, or expectations.

For example, a logic model to inform the design and evaluation of a faculty advancement initiative focused on improving research success and inclusive excellence could identify specific components that include:

1. **Environment**
  - Existing faculty needs (e.g., mentoring)
2. **Inputs**
  - Institutional resources available to address those needs
3. **Activities**
  - Use and implementation of institutional resources to support faculty development
4. **Outputs**
  - Short-term indicators: mentor-mentee matching, completion of Individual Development Plans (IDPs)
  - Intermediate outcomes: NIH grant submissions, peer-reviewed publications per year
  - Long-term outcomes: extramural funding success, faculty retention, increased diversity in leadership roles

Equity-focused metrics could be embedded across the model activities and evaluation, including tracking faculty achievements by gender, race/ethnicity, and academic rank.

**Figure 2.**
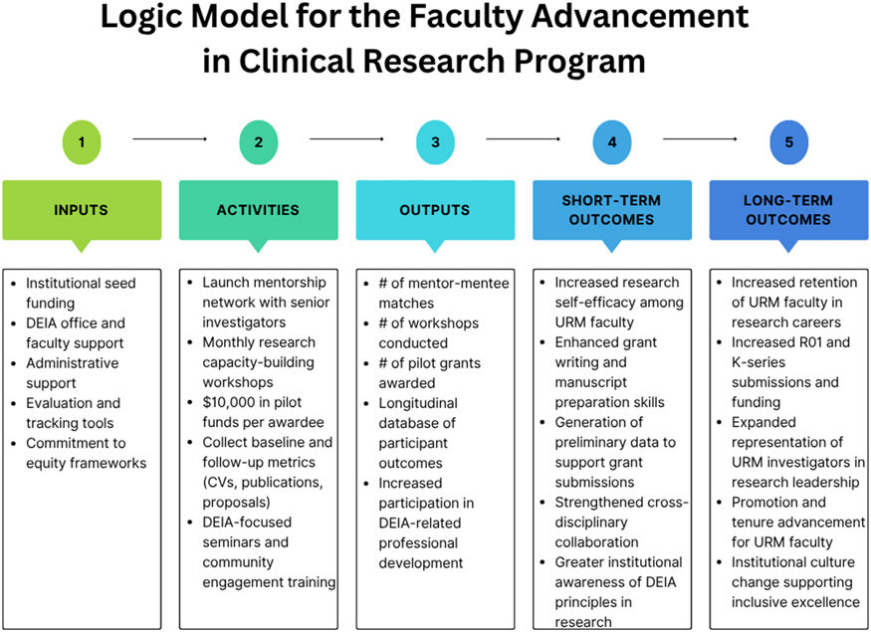
Logic model for the Faculty Advancement in Clinical Research Program. Source: Developed by study team (self-generated).

Major findings of using this model may include the identification of potential barriers (i.e., low rates of participation in grant-writing workshops or low satisfaction with mentoring received among women and URM faculty). These findings could inform development support strategies such as the implementation of more flexible formats and additional support services (e.g., dependent care stipends) or mentor-specific training on mentoring and culture/gender-sensitive communication. These adaptations would increase faculty engagement and NIH application rates among URM investigators, demonstrating the utility of logic models in producing timely, data-informed course corrections to promote equity and effectiveness (see Figure 2).

While logic models emphasize the relationships and pathways between existing resources, proposed activities, and expected outcomes, theory-derived evaluation models, such as the Realist Framework among others [24–26], focus on understanding the mechanisms by which programs operate, their target populations, and the contexts in which they are effective [27]. The Realist Framework enables evaluators to explore complex pathways between program components, moving beyond simple cause-and-effect relationships. It identifies several critical aspects: (a) the specific context and conditions affecting program implementation and outcomes; (b) the processes or program mechanisms generating outcomes; (c) the results of the program, including positive or negative, and short-or long-term effects; and d) how these mechanisms function within particular individual or institutional contexts result in the specific outcomes.

For example, to complement the faculty development program logic model described in Figure 2, the Realist Framework could be used to deepen the evaluation of the short- and long-term outcomes by identifying why specific components of the program are more effective for certain subgroups and under what conditions. If the study results show that URM faculty with caregiving responsibilities report lower participation in grant writing workshops, applying a Context-Mechanism-Outcome (CMO) lens can reveal the following configuration: (a) **Context**: URM faculty balancing caregiving duties; (b) **Mechanism**: Introduction of flexible scheduling and dependent care stipends increased perceived accessibility and motivation; and (c) **Outcome**: Improved engagement in grant preparation and increased grant submission rates among URM faculty. This analysis not only explains differential workshop participation in URM faculty balancing caregiving duties but can also guide targeted adaptations to the faculty development program to promote equity in access, engagement, and outcomes.

The integration of the Realist Framework with the logic model can provide a more robust evaluation strategy, ensuring alignment with intended goals while uncovering the mechanisms that drive effectiveness in diverse faculty populations. Together, these frameworks can support iterative improvements and strengthen programs’ impact on inclusive faculty development and success.

### Data collection, tracking, and accountability mechanisms

Inadequate data collection, tracking, and accountability mechanisms in the evaluation of equity and inclusion initiatives can significantly impair the effectiveness of equity and inclusion efforts and resource allocation. Clear benchmarks and measures are crucial in evaluating equity and inclusion programs. Establishing standardized data protocols, performing regular audits, and integrating real-time reporting systems ensures data integrity and consistency in tracking progress. Data governance policies, privacy protections, and varying definitions of demographic categories must be negotiated across units. Senior leadership, including chief information officers, chief diversity officers, and institutional research directors, must collaborate to establish data-sharing agreements, standardized definitions, and clear accountability structures. A key component of this approach is leveraging both internal and external data sources. Internal data from human resources systems, employee surveys, and performance evaluations provide critical insights into organizational culture, recruitment patterns, retention rates, and career advancement across demographic groups. Meanwhile, external data sources – including industry standards within academic medicine, healthcare workforce reports, and datasets from the (NIH) and the Association of American Medical Colleges – offer critical context for assessing DEIA efforts. By combining these datasets, organizations can better accurately evaluate their inclusive landscape, identify gaps, and align their strategies accordingly.

At the national level, government-led initiatives are developing equity and inclusion dashboards to provide comprehensive data visualization tools that monitor and evaluate equity and inclusion efforts across various sectors [28]. These dashboards aggregate key metrics and present them in an accessible format, promoting transparency, accountability, and data-driven decision-making. The information from these dashboards can be used not only for evaluation but also to track trends, identify disparities, and guide strategic planning for resource allocation. Simultaneously, at the institutional level, initiatives like the collaboration between the University of Michigan Library and the National Center for Institutional Diversity (NCID) are driving innovation through the development of an open data toolkit [29]. This toolkit is designed to empower researchers, foster collaboration, and drive positive social change by providing tools for navigating complex data and promoting data-driven insights into equity and inclusion issues.

Organizations can also enhance data collection by integrating more technically advanced approaches to evaluation, such as Natural Language Processing (NLP) and machine learning, which are undoubtedly powerful tools to process qualitative and observational data. NLP allows organizations to analyze open-ended feedback from employees, social media, or public forums, providing a deeper understanding of attitudes toward equity and inclusion. For example, during the COVID-19 lockdown, researchers utilized NLP to assess public sentiment regarding equity and inclusion in transportation systems by mining social media data from several New York City counties [30]. This method offers a more dynamic and real-time assessment compared to standard surveys, enabling institutions to respond quickly to emerging issues.

Dashboards, which are data visualization tools that consolidate metrics into a comprehensive interface, can be more transformative by integrating predictive analytics to forecast outcomes based on current data trends. This forward-looking approach enables organizations to identify potential problem areas and prevent them before they manifest. Effective data tracking in equity and inclusion involves more than just measuring outcomes; it requires consistent monitoring of the actions and processes driving those outcomes. Organizations can track progress and adjust strategies by establishing regular review cycles where data is evaluated against the predetermined goals. This iterative approach not only ensures that equity and inclusion initiatives stay aligned with larger organizational objectives but also provides valuable insights into which specific strategies are advancing or hindering equity and inclusion related progress.

### The value of data-driven approach in evaluating equity and inclusion efforts

A data-driven approach is essential for evaluating equity and inclusion initiatives, ensuring that assessments are comprehensive, rigorous, and actionable. From 2011 to 2015, a medical school demonstrated the effectiveness of such an approach in fostering cultural competence. Through 13 voluntary events attended by 562 participants, the initiative used a balanced data collection strategy that incorporated both Likert scale ratings and open-ended questions, ensuring a more nuanced analysis by blending quantitative and qualitative data [31]. This balanced approach avoided a one-dimensional evaluation, ensuring a comprehensive analysis by incorporating both quantitative and qualitative data. Over-reliance on qualitative data alone can limit the depth and breadth of the evaluation, potentially overlooking important quantitative insights that could provide a more complete understanding of the initiative’s impact.

Integrating robust data analytics, such as advanced statistical analysis, predictive modeling, and trend identification, improves the validity, reliability, and generalizability of findings [32]. Applying quantitative frameworks allows evaluators to uncover patterns and disparities that may not be immediately apparent. For example, the Coordination and Evaluation Center of the Diversity Program Consortium, funded by NIH demonstrates the effectiveness of a multilevel evaluation strategy that incorporated both demographic trends and outcome tracking over time to assess the impact of structured research training on underrepresented students [33]. Their approach combined quantitative outcomes (e.g., research engagement, degree progression) with institutional-level data to identify key leverage points for improving diversity [33]. These methods provide more precise identification of areas needing improvement and the development of evidence-based strategies to promote equity and inclusion.

A commitment to rigorous, data-informed evaluation enhances the accuracy and relevance of assessments while also fostering innovation, accountability, and sustained progress in equity and inclusion initiatives across sectors.

## Conclusion and future directions

Underrepresented groups face systemic barriers within academic medical institutions in the U.S. that hinder research productivity and career advancement. Efforts to promote equity and inclusion are further undermined by fragmented evaluation approaches, the lack of guiding theoretical frameworks, inadequate data collection and accountability, and insufficient data-driven strategies, highlighting the need for comprehensive institutional reforms.

Future advocacy efforts in fostering equity and inclusion within workplace cultures must take on both entrenched institutional barriers and a shifting federal landscape. Recent federal rollbacks on diversity initiatives present a significant existential challenge, threatening to cut off resources, diminish accountability mechanisms, and discourage the open promotion of equity-related goals [34,35]. These policy shifts may disincentivize institutions from prioritizing equity and inclusion programs. Additionally, they potentially create a chilling effect on efforts aimed at dismantling systemic racism, promoting health equity, and improving representation across the biomedical workforce [35,36]. In response, academic medical institutions must develop resilient, evidence-based equity frameworks that can withstand shifts in federal support. This requires strong internal infrastructure, strategic alignment, and leadership that champions and appropriately invests in data-driven evaluation. Advocates play an essential role in incentivizing institutional leaders and key decision makers to endorse and implement recommendations for change. As external political climates evolve, institutional commitment and internal accountability will be key to preserving momentum and ensuring that efforts to advance equity remain both meaningful and measurable.

In summary, while challenges that are both internal and external to academic medical institutions remain, the path forward for organizations committed to equity and inclusion involves rigorous, multifaceted evaluative approaches [37]. This commentary underscores the importance of applying such approaches if we are to truly promote transformative progress in this area. Furthermore, continued dissemination of holistic and data-driven evaluative efforts is needed if we are to continue to elevate scholarly discourse and build a strong evidence base of best practices for advancing equity and inclusion that can effectively guide the broader academic medical community.
