# Evaluating efforts to advance equity and inclusion in academic medicine: Charting a path for transformative change – CORRIGENDUM

**DOI:** 10.1017/cts.2026.10731

**Published:** 2026-03-25

**Authors:** Ju-Hsin Chen, Emma K.T. Benn, Erin Brittain, Nihal E. Mohamed

When this article was published in the Journal of Clinical and Translational Science, it listed the authors in an incorrect order. Nihal Mohammad has now been moved to the end of the author list so that their status as the senior author is reflected.
